# The bacterial microbiome and resistome of house dust mites in Irish homes

**DOI:** 10.1038/s41598-024-70686-y

**Published:** 2024-08-23

**Authors:** Amal Aljohani, David Clarke, Miriam Byrne, Gerard Fleming

**Affiliations:** 1 Microbiology, School of Biological and Chemical Sciences, University of Galway, Galway, Ireland; 2 Bat Conservation Ireland, Dublin, Ireland; 3 Physics, School of Natural Sciences, University of Galway, Galway, Ireland

**Keywords:** Dust mites, Resistome, Antibiotic resistant genes, Dust samples, qPCR SmartChip, 16 S rRNA microbiome, Microbiology, Environmental sciences

## Abstract

Dust samples were collected from Irish homes. House Dust Mite and storage mites were separated from the dust. The microbiome and resistome of mites and originating dust were assessed using a culture-independent approach. The bacterial microbiome of mites and dust were predominantly populated by Staphylococci. There was a highly significant (*P* = 0.005; Spearman’s rank test) correlation between the bacterial microbiome of mites and the dust. One-hundred and eighteen antimicrobial resistance genes (ARGs) were associated with mites and 176 with dust. Both contained ARGs encoding resistance for multi drug resistances, macrolide-lincosamide-streptogramin B, mobile genetic elements, Beta-lactam, Tetracycline and Aminoglycosides. By contrast, 15 ARGs were found for a laboratory-grown strain of *Dermatophagoides pteronyssinus*. A significant difference (*P* = 0.03; t test) was found in means between the resistome of mites and the household dust from which they emanated. No significant correlations (*P* = 0.23 and *P* = 0.22; Mantel test) were observed between the microbiome and resistome of mite and dust samples. There was not a significant difference (*P* = 0.54; t-test) between the means of ARGs for homes with and without a history of antibiotic use.

## Introduction

As a species, humans spend more than 90% of their time in Indoor environments (homes, offices, hospitals, schools and hotels^1,2^. The quality of air in the indoor environment impacts on human health (EPA’s indoor air quality website; https://www.epa.gov/report-environment/indoor-air-quality). Exposure to microorganisms^3^, pets^4^, biotic and abiotic dust^5^ and chemicals^6^ may adversely affect health, and exposure to these agents can lead to allergic diseases^7^.

House dust mites (HDM) are microscopic insects belonging to Acari. Acarines are represented by three suborders of acariforms (Astigmata, Prostigmata and Oribatida). They inhabit moist and warm areas of the indoor environment (e.g. mattresses and soft furnishings^8^. The European *D. pteronyssinus* is the most common HDM in Europe^9^. They feed on skin shed from humans and animals^8^. The exoskeleton of live and dead mites and their detritus can result in sensitization of the human and animal immune system resulting in asthma and dermatitis^10,11^. It is estimated that these allergic diseases affect approximately 65–130 million individuals on a global scale^12^.

Bacteria and fungi are associated with the HDM microbiome, whether that of the exoskeleton, gut contents and dander^13–15^. The microbiome is relatively well investigated and it is known that the microbiome is mainly characterised by Gram-positive bacteria and fungi^16–18^. Results of studies with laboratory-reared mites showed that a symbiotic relationship existed between microorganisms and mites which facilitated the mites’ digestion process^19^.

The spread of antibiotic resistance is of global concern. Household insects such as bed bugs, cockroaches can harbour microorganisms that carry antimicrobial resistance genes (ARGs), and in certain instances, express these genes, resulting in antimicrobial resistance (AMR)^20^. It is known that bacteria associated with fish faeces/marine sediments^21^and house flies^22^ can carry genes for AMR. The incorporation of oxytetracycline to the feedstuff of dust mites can result in a significantly altered gut microbiome which varies from species to species^23,24^. To date, there are no reports in the scientific literature which details the link between the microbiome and resistome of environmentally-sampled dust mites and their detritus as a component of household dust.

In view of the above knowledge gap, the aims of the present study are to (a) Investigate the bacterial microbiome of household-sampled dust mites and dust using culture-independent approaches. (b) Determine if household-derived mites and dust carry antibiotic-resistance genes and establish the relationship between both elements in the absence and presence of antibiotic use in the household.

## Results

Table 1 summarises the mite species identified from homes 1–6, the source of the mites (carpet or mattress) and whether antibiotics were used in the household in the past 6 months. The European mite—*Dermatophagoides pteronyssinus* was the dominant species in three of the homes sampled. *Glycyphagus domesticus* is a storage mite (Home 1) and *Lepidoglyphus destructor* is a predator mite. D denotes the dust from which the mites were isolated.

**Table 1:** House dust mite and dust samples from Irish homes and laboratory strain (Galway) n = 12: 1–6 represented mite samples and 1D–6D represented dust samples, 4 and 4D showed the laboratory strain (control).

Sample ID	Home	Mite species	Source	Antibiotic use
1	Home 1	*Glycyphagus domesticus*	Carpet	No
1D		Dust and mite debris		
2	Home 2	*Dermatophagoides pteronyssinus*	Carpet	No
2D		Dust and mite debris		
3	Home3	*Dermatophagoides pteronyssinus*	Mattress	Yes
3D		Dust and mites debris		
4	Control	*Dermatophagoides pteronyssinus*	Laboratory strain	No
4D		Rearing Diet and mites faeces		
5	Home 5	*Dermatophagoides pteronyssinus*	Mattress	Yes
5D		Dust and mites debris		
6	Home 6	*Lepidoglyphus destructor*	Carpet	Yes
6D		Dust and mite debris		

### The bacterial microbiome of mites and dust samples

**Fig. 1:**
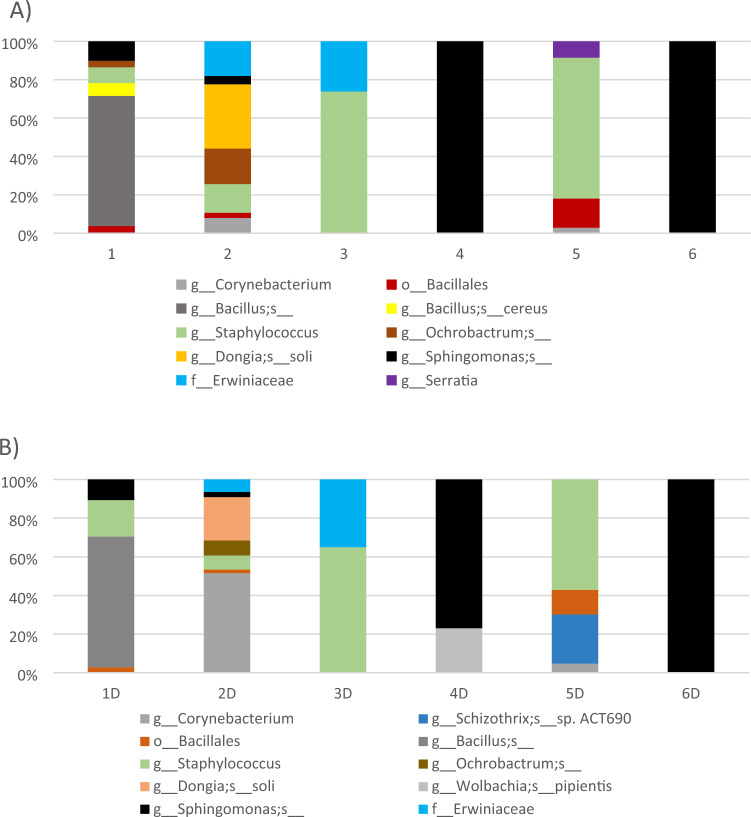
The ten most abundant bacterial species found for dust mite and household dust samples (n = 12). Samples 4 and 4D represented the laboratory strain (control).

In this study, a culture-independent approach was taken to identify the bacterial microbiome of homogenised mites and the dust from which they were emanated. Illumina MiSeq was used for sequencing of 16S rRNA from the bacterial microbiota. A highly parallel qPCR array with 384 primer sets were used for the detection and quantification of ARGs, other genes encoding resistance to antibacterial compounds, mobile genetic elements MGE associated genes, and the 16S rRNA. The qPCR array was used to analyse the composition of dust mite resistomes and environmental dust from Irish homes were also analysed to detect the possible risk of ARGs spread indoor environments. The ten most abundant bacterial species identified from sampled dust mites (DM) and dust (D) samples is represented in Fig. 1. Samples were predominantly populated by Gram-positive microorganisms of which Staphylococci were most abundant for household mite and dust samples. Samples 4 and 4D represented the control (laboratory strain *D. pteronyssinus*) which had a less diverse bacterial microbiome and *Sphingomonas sp* were dominant for mite and dust samples. The microbiome profile associated with the control strain (and feedstuff) were similar to that of the storage mite (*Glycyphagus domesticus* and *Lepidoglyphus destructor*) and the dust samples taken from Home 6.

### Resistomes of HDM and dust samples

**Fig. 2:**
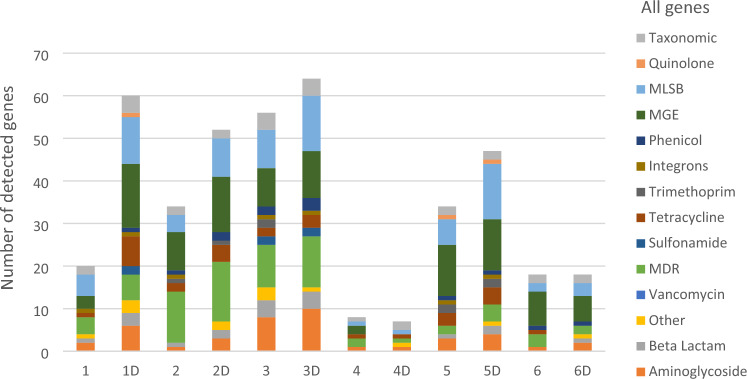
Number of detected genes in environmental HDM, dust samples (n = 12) according to different antibiotic classes: 1–6 represented mite samples and 1D-6D represented dust samples, 4&4D showed the laboratory strain (control).

The resistome for identified mites and the dust from which they emanated was examined. Details are provided in Table 1. The samples chosen for analysis were based on the nature of biotopes, the source of the dust (carpet or mattress), and whether there was a history (or not) of antibiotic use in the home. The number of detected genes in each individual sample is represented in Fig. 2. From 384 genes analysed from different antibiotic classes, a total of 118 and 176 resistant genes were detected in mite and dust samples respectively. These genes encoded for resistance to different antibiotic classes. These include; Multi-drug resistance (MDR), macrolide-lincosamide-streptogramin B (MLSB), mobile genetic element (MGE), Beta-lactam, Tetracycline and Aminoglycosides detected in all mite containing samples. The profile of ARGs, whether it is from mite or dust, appears to be domicile specific (Fig. 4) but were similar for dust and mites for each individual home.

**Fig. 3:**
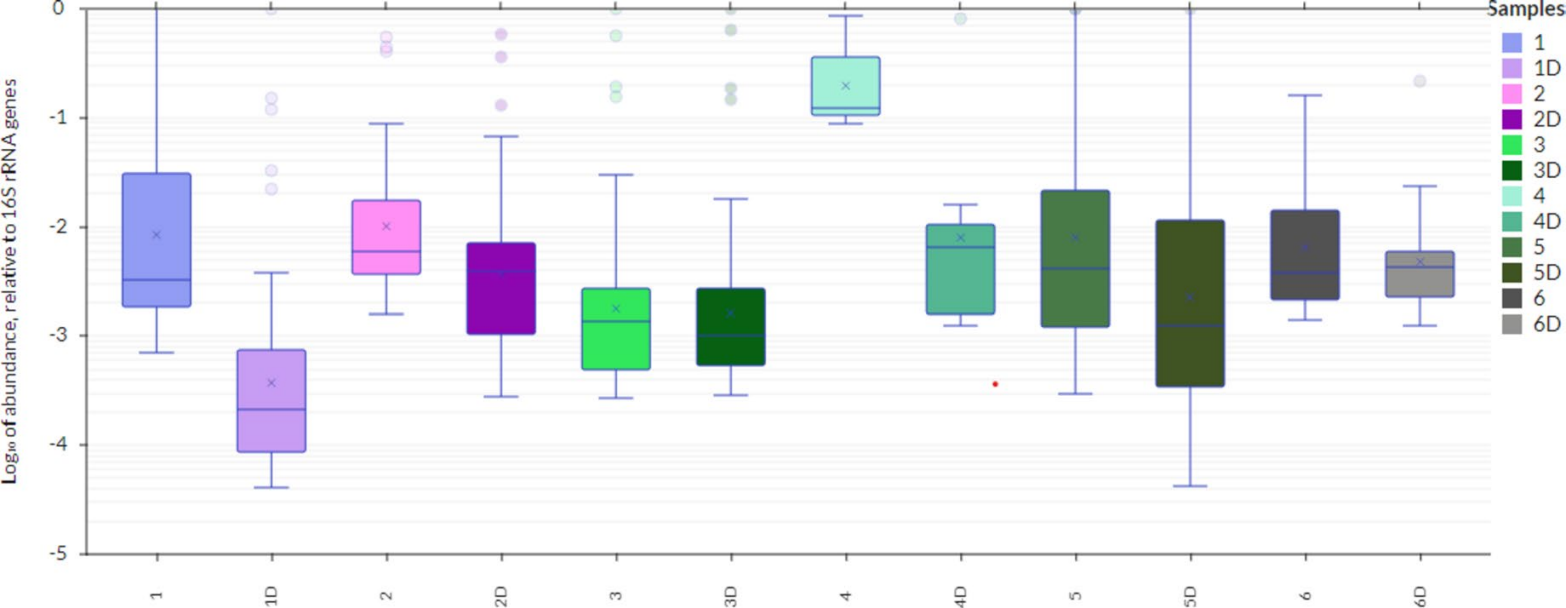
Relative abundances of the genes detected in the HDM in proportion to the 16S rRNA gene (as log values) in mite and dust samples (n = 12). 1–6 represented mite samples and 1D–6D represented dust samples, 4&4D showed the laboratory strain (control).

**Fig. 4:**
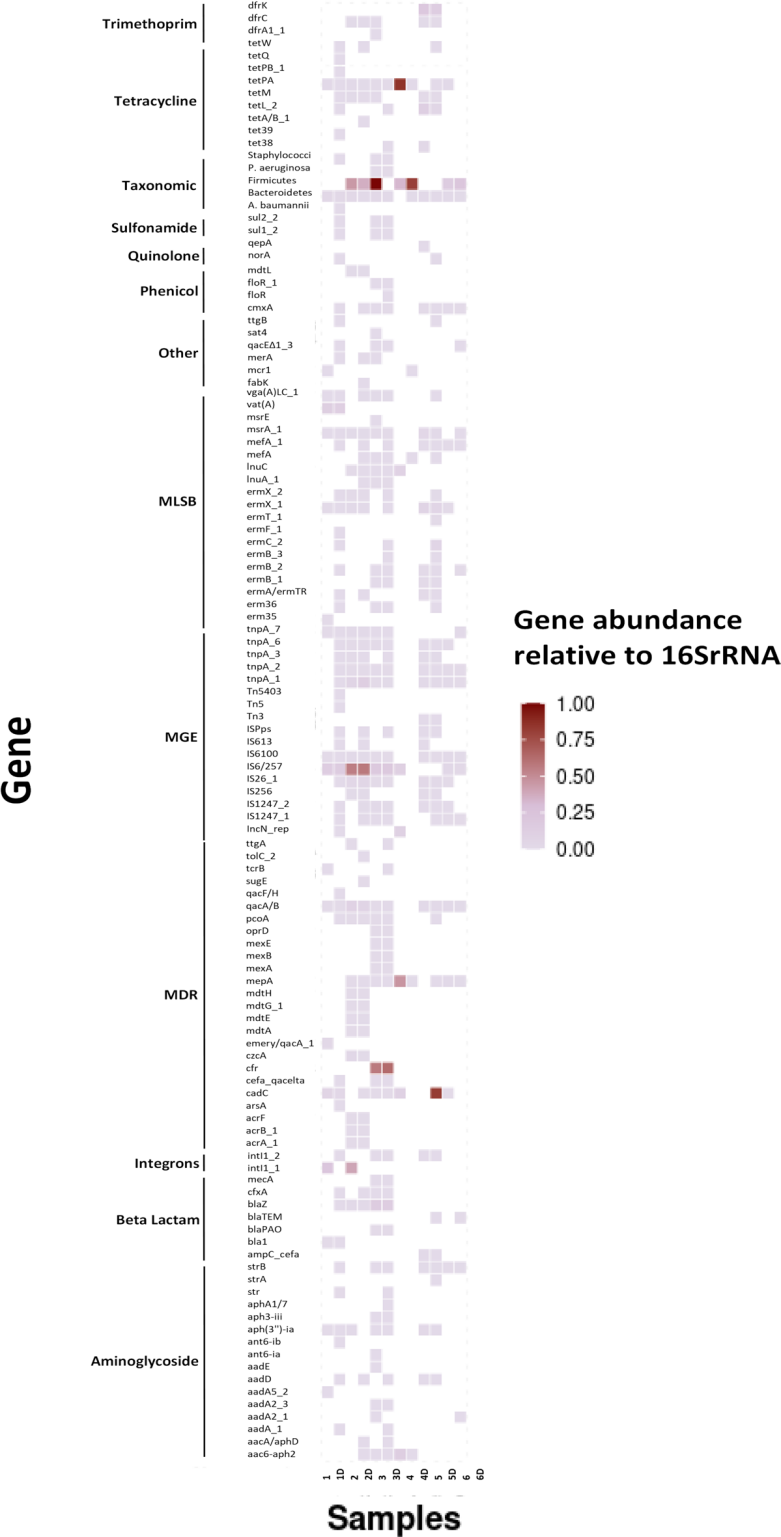
Resistome composition of HDM and dust in Galway homes (n = 12), Ireland. The colours in the heatmap show gene abundances relative to 16S rRNA gene. The X axis represents the sample ID and the Y axis represents the antibiotic group. 1–6 represented mite samples and 1D–6D represented dust samples, 4 and 4D showed the laboratory strain (control).

Fifteen antibiotic resistant genes were identified in the laboratory-reared strain of *D. pteronyssinus* (FERA, UK Ltd). This was maintained under aseptic conditions, and essentially axenic. The profile of these ARGs (MGE, MDR, Tetracycline, MLSB and Aminoglycosides) were different to those of the sampled mites and associated dust e.g. Firmicutes, Bacteroides, *IS6/257- IncN_rep, cadC- mepA, tetPA, InuC, aac6-aph2*. The profile for mite and dust were similar. The relative abundance of the genes detected in each home as a proportion of 16S rRNA in mite samples and dust samples varied from 10^–3^ to 10^–1^ (Fig. 3). Heatmap analysis showed that Firmicutes and Bacteroides genes were predominant in most (5 samples) of mite and dust samples (Fig. 4).

Multidrug efflux resistant genes (*cadC*, *cfr* and *mepA*) were also detected in mites and dust associated with samples 3 and 3D samples and in the control strain sample 4 (Fig. 4). Gene IS6/257, a known mobile genetic element^25^, was also found in 11 samples out of the 12 samples analysed.

Statistical analysis (t-test) showed that there was not a significant difference between the means of the number of detected genes for mite and dust samples irrespective of whether antibiotics were used (*P* = 0.54) or not used (*P* = 0.43) in the preceding six months. Furthermore, the number of resistance genes detected did not significantly differ for biotope-associated mites (*P* = 0.35) or dust (*P* = 0.47).

Whist *D. pteronyssinus* was the dominant mite species detected in this study, two homes presented with storage mites *Glycyphages domesticus* and *Lepidoglyphus destructor* (Table 1). There was no significant difference between the number of resistance genes connected with these species (*P* = 0.09: t test) and the associated dust (*P* = 0.84: t test).

**Fig. 5:**
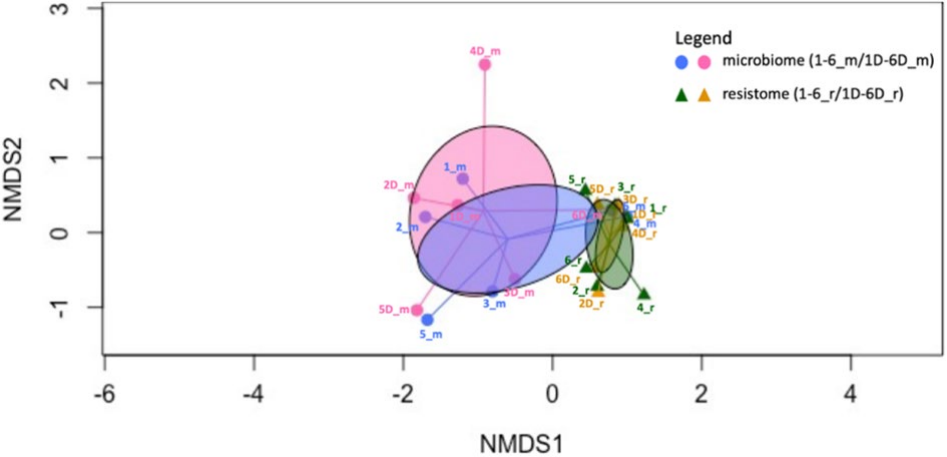
NMDS ordination of microbiome and resistome of mite and dust samples. Circles show 95% confidence area for standard error of the centroids of the microbiome and resistome in different sample types. 1-6D_m and 1-6_m = microbiome for dust and dust mite for samples (n = 12), respectively. 1-6D_r and 1-6_r = resistome for dust and dust mite for samples (n = 12), respectively.

The similarity of the ARGs between mites and dust indicates a homogeneity of environment. Ordinate (Principle Component Analysis) was employed to ascertain the degree of relationship between the microbiome and resistome of dust and mite samples. Data are presented in Fig. 5. There is significant overlap between the microbiome and resistome for both dust mite and dust samples. The microbiome of dust and the associated mite population is greatly similar (correlated), and this correlation was highly significant (ρ = 0.5842, *p* = 0.005). Additionally, a significant correlation (ρ = 0.6357, *p* = 0.03) was found for the resistome of dust and dust mites. Furthermore, the Mantel test showed a positive correlation between the mite and dust microbiome, r = 0.584 and between the mite and dust resistome r = 0.635.

## Discussion

The similarity/dissimilarity between the microbiomes for dust mites (exoskeleton and gut combined) and the dust from which they were sampled in domestic settings is unknown. Furthermore, the presence and magnitude of Antimicrobial Resistance Genes (ARG) for mites and their associated household dust is unreported in the scientific literature. The first aim of this undertaking was to investigate the relationship between the bacterial microbiome of house dust mites (including storage mites) and that of the dust from which they emanated. Whilst the microbiome for laboratory-reared dust mites is well established, particularly for associated fungi^17^, mite-associated microbiomes can vary with environmental conditions in which mites are reared in the laboratory^14,17,26^. In the present study, it was shown that the bacterial microbiome of sampled mites from domestic settings were predominantly enriched with Gram positive-Staphylococci (132 OTU) and to a lesser extent *Corynebacterium, Schizothrix, Bacillus, Ochrobactrum, Dongia, Wolbachia, Sphingomonas, Erwiniaceae, Ochrobactrum* and *Serratia* (Fig. 1). Others have found that Staphylococci were most abundant in the gut contents of laboratory-reared *D. pteronyssinus*^27^. *Staphylococci* are commensal with human skin, transferrable between individuals and are present in dander^28^. In this study, storage mites were found in two homes, and the most abundant bacterial species in those homes were *Bacillus* and *Sphingomonas* (Table 1). Laboratory-reared storage mites appear to have a different microbiome to that of *D. pteronyssinus* and inclusive bacterial species such as *Cardinium* and *Wolbachia*^16^. The microbiome of environmentally-sampled HDM and storage mites are different (Fig. 1) and it was observed that the microbiome of the storage mite *Lepidoglyphus destructor* showed stronger similarity to that of the laboratory-reared *D. pteronyssinus* than to those mites sampled from the home. The abundance of the lesser-dominant bacterial species differed for each home (Fig. 1) and as such, may have different microbiomes. For example, home 1 was dominated by *Glycyphagus domesticus* and home 2 by *D. pteronyssinus*. The microbiomes of dust samples also differed depending on whether they were predominantly populated by dust mites or storage mites. The household dust associated with *D. pteronyssinus* contained signatures for *Staphylococci*, *Corynebacterium, Schizothrix, Bacillus, Ochrobactrum, Dongia, Wolbachia and Sphingomonas.* Dust containing storage mites on the other hand were predominantly populated by *Sphingomonas*, *Staphylococci* and *Bacillus*. Others have reported that the microbiome associated with household dust was rich in *Proteobacteria* and to a lesser extent *actinobacteria*^26^. Actinobacteria were detected in household dust by others^29,30^ but were not detected in the household dust in the present study. The nature of the dust microbiome may depend on aetiological inputs. The dust sampled from homes where inhabitants reported incidences of asthma, atopy or hay fever was populated by Cyanobacteria, Bacteroidetes, and Fusobacteria whilst dust from homes with lesser incidences of asthma in particular contained mainly Firmicutes^31^. The microbiome associated with household dust is not universally homogenous and has many inputs which is reflected by the diversity of the microbiomes between studies. A larger study is needed to determine if environmental factors such as relative humidity, number of inhabitants, presence of pets, age of home etc. has a bearing on the dust and mite-associated microbiome.

In the present study, the laboratory-reared strain *D. pteronyssinus* (FERA UK LTD) was predominantly populated by *Sphingomonas sp* (Fig. 1), as was the feed source in which it was grown. Whilst Gram-positive microorganisms are dominant among the laboratory-reared strains, there is considerable variation at the species level. For example, the Czech strain of *D. pteronyssinus* was reported to harbour *Kocuria* and *Staphylococci* whilst the Korean variant were dominated by *Klebsiella pneumonia* and Rhizobium taxa^14^. It is apparent that there is a variation at species level even when dust mites are reared under sterile conditions to promote an axenic state. This may be attributed to slight differences in rearing conditions in the food stock used to rear the mites.

Insect and animal-borne ARGs are of concern since these can act as reservoirs for antimicrobial resistance to antibiotics^20^. Here, 118 resistant genes were detected for mite samples from a diverse grouping of antibiotic classes (Fig. 2). Each sample had its own unique resistome but firmicutes genes were the most abundant gene in most mites and dust samples as shown in (Fig. 4). The diversity of ARGs is large, and may reflect the selection pressures within the domains for the maintenance of such genes (e.g. presence of pets, number of inhabitants etc.). Interestingly, MDR, MLSB, MGE, Beta-lactam, Tetracycline and Aminoglycosides antibiotic classes were detected in all mites and these are acknowledged as being clinically-important and universally-prescribed antibiotics^32^. Tetracyclines are commonly used in veterinary and human medicine^33^. Here, *tet*PA and *tet*M genes that belong to tetracycline class were detected in most mite and dust samples. Carbapenems (Beta-lactam) are of broad spectrum and used to treat Gram-positive and Gram-negative infections. Furthermore, mobile Genetic Elements (MGE) enable exchange of genetic material between bacteria^34^. MGE genes were detected in mite and dust samples in particular: *Is6/257* and *IncN_rep*. (Fig. 4). The mechanism of MGE is to acquire the resistant genes from environment via horizontal gene transfer^20^.

Household dust samples were represented by 176 resistant genes (Fig. 2). Dust resistomes were enriched with ARGs, such as firmicutes, Macrolids-lincosamide-streptomycin B (MLSB) and tetracycline (Fig. 4). Others found that dust resistome was enriched with ARGs such as *tet*(W), *blaSRT-1*and *erm* (B) and these were associated with the presence of antimicrobial chemicals such as triclosan in the dust microbiome^35^.

The laboratory-reared strain (*D. pteronyssinus*: FERA, Ltd UK) and associated feed stuff had the least diverse and the lowest abundance of antimicrobial resistance genes (Fig. 2). The detected gene were 8 (mites only) and 7 genes from the (reared diet and debris). This may be due to the axenic nature of the mites and aseptic handling during the rearing process^8,17^.

The second aim of this study was to investigate the correlation between microbiome/resistome of mites and dust. The association between the microbiome of mites and dust samples from which they emanated was highly significant (*p* = 0.005: Spearman’s rank correlation). This is not surprising in that they share a common environment and both entities interact with each other. The relationship between the abundance of antimicrobial resistance genes for sampled mites and dust (Fig. 4) and ordinate analysis of the same (Fig. 5) shows that the resistomes of the mites and the dust (from which they emanated) was similar and independent of the home from which they were sampled. There is likely significant interaction between mites, their detritus and the natural microflora found in household dust samples. This interaction is likely to be at the gut level but also mediated by the carriage of microorganisms carried on the mite exoskeleton.

Antibiotic use promotes the presence and persistence of ARGs in the environment^36^. Antibiotic use was recorded for three homes (Homes 3, 5 and 6) in this study whilst the remaining homes had no history of use (Table 1). There was no significant difference in the mean of the number of genes detected between mite and dust samples from homes with/without antibiotic use (*P* = 0.54 and *P* = 0.43 at the 5% level). Whilst, a larger sample size would have been desirable, (and in particular, an even split for homes with or without a history of antibiotic use and the explicit delineation of the antibiotic used), the present findings indicate that antibiotic use in the home did not significantly alter the resistome of domestically—associated mites.

In conclusion, the relationship between the microbiome and resistome of the mite and dust samples taken from Irish homes is reported upon. The similarity of the microbiome and resistome suggest that there is significant interaction between these milieus with the potential for the transfer of ARGs. Foremost this is a reservoir for AMR that has not been previously recognised. As with any reservoir for AMR, it warrants monitoring, particularly to determine the degree of horizontal gene transfer of AMR in the home. A larger study for rural and urban environments might be appropriate. To the authors’ knowledge, this is the first report of such an association outside that of the laboratory-reared mite setting.

## Materials and methods

### Dust sampling, mite isolation and identification

Dust samples were collected from fifty-six homes (mainly student dwellings) in Galway, Ireland. Sampling was carried out over a two year period (2019–2021) encompassing all seasons. Dust was gathered from floors, mattresses, carpets, soft furnishings or pet bedding. A SKC Flite-2 air sampling pump (flow rate in l/min = 13.68 ± 0.03 S.D.) fitted with a 75% (w/v) alcohol disinfected and dried cassette containing a pre-sterilised Whatman Nucleopore Polycarbonate membrane filter (diameter 37 mm, pore size 0.4 µm) was used for sampling^37^. A surface area of 2500 cm^2^ was sampled using the protocol described previously^38^. Cassettes containing dust were stored in a refrigerator and transferred to the laboratory within 24 h of sampling.

Of the fifty-six homes sampled, five homes presented with a sufficient mite density (10 mg of mites) to enable DNA extraction. These samples were taken from carpets or bedding (Table 1). Of the five homes with 10 mg of mites or greater, the inhabitants of three of these homes indicated they had used antibiotics in the previous 6 months.

Mites were manually extracted from the dust using a sterile fine-bristle brush and a 10 µl plastic inoculation loop viewed under 40× magnification (Olympus stereomicroscope) and transferred to a pre-weighed sterile petri dish which was sealed with tape to prevent the escape of mites before further treatment. Mites separated from household dust were viewed under 100× magnification (Olympus, SZ30) and identified to species level with the aid of the identification keys of^8,39^.

Laboratory-reared *D. pteronyssinus* (10 mg) served as axenic controls for microbiome and resistome analysis, as did the growth media used for their rearing. In total, 24 samples, representing mites, the dust from which they were isolated, a history of use/non-use in the home and axenic mite/growth medium controls were presented for microbiome and resistome analysis.

### DNA extraction from mites/dust/growth media

DNA extraction was performed using the PowerSoil Pro kit (Qiagen). The DNA quality and concentration were analysed with a Nanodrop 1000™ spectrophotometer (Thermo Scientific, Wilmington, DE, USA) and pre-treatment using the FastPrep method (MP Biomedicals, Irvine, CA, USA) following the manufacturer's instructions. DNA extraction from HDM, house dust and mite-rearing media (microbiome analysis) was undertaken by Eurofins, Germany and extraction for resistome analysis was carried out by Resistomap, Finland.

### Microbiome analysis—bacterial 16S rRNA and bioinformatics

For investigation of bacterial microbiome from HDM, dust samples and mite-rearing media; the extracted DNA was sequenced using the Illumina MiSeq Personal Sequencer target the region V3–V5 of the 16SrRNA for the bacterial microbiome Eurofins, Germany. We employed a primer pair 515F (GTGCCAGCMGCCGCGGTAA) and 806R (GGACTACHVGGGTWTCTAAT). PCR thermal procedures were as follows with 35 cycles for amplification of bacteria: initial denaturation for 5 min at 95 °C; 35 cycles of 30 s at 95 °C, 30 s at 55 °C, and 45 s at 72 °C; and a final extension of 10 min at 72 °C. Moreover, negative controls including no-template (three replications) and template from un-used swabs (three replications) were also subjected to amplification^26^. Products were confirmed by gel electrophoresis.

As a first step of the microbiome analysis, all reads with ambiguous bases (“N”) were removed. Chimeric reads were identified and removed based on the de-novo algorithm of UCHIME^40^ as implemented in the VSEARCH package^41^.The remaining set of high-quality reads was processed using minimum entropy decomposition^42,43^. Minimum Entropy Decomposition (MED) provides a computationally efficient means to partition marker gene datasets into OTUs (Operational Taxonomic Units). Each OTU represents a distinct cluster with significant sequence divergence to any other cluster. By employing Shannon entropy, MED uses only the information-rich nucleotide positions across reads and iteratively partitions large datasets while omit ting stochastic variation. The MED procedure out performs classical, identity based clustering algorithms. Sequences can be partitioned based on relevant single nucleotide differences without being susceptible to random sequencing errors. This allows a decomposition of sequence data sets with a single nucleotide resolution. Furthermore, the MED procedure identifies and filters random “noise” in the dataset, i.e. sequences with a very low abundance (less than ≈ 0.02% of the average sample size).

To assign taxonomic information to each OTU, DC-MEGABLAST alignments of cluster representative sequences to the sequence database were performed. A most specific taxonomic assignment for each OTU was then transferred from the set of best-matching reference sequences (lowest common taxonomic unit of all best hits). Hereby, a sequence identity of 70% across at least 80% of the representative sequence was a minimal requirement for considering reference sequences. Further processing of OTUs and taxonomic assignments was performed using the QIIME software package (version 1.9.1, http://qiime.org/). Abundances of bacterial taxonomic units were normalized using lineage specific copy numbers of the relevant marker genes to improve estimates^44^.

### Resistome analysis by SmartChip qPCR

The quantification and abundance ARGs, integrons and MGEs and 16S rRNA gene in each sample were analysed using customized primer sets as described and validated previously in^21,45^ in a high throughput method, SmartChip qPCR system (WaferGen Biosystem, Freemont, CA, USA). The reaction was carried out on the Takara SmartChip Real-time PCR system. Briefly, 384 genes were investigated, with each smartChip qPCR containing 5184 reactions with 100 nL volume for each wells. All samples were performed in triplicate.

### Statistical analyses of data

The differences between the means of the numbers of antibiotic resistant genes for HDM and dust samples was assessed using the Student T-test (Minitab, 2019). This test was also used to ascertain the significance difference of means between the numbers of antibiotic resistant genes, antibiotic usage, mite species and source of samples (carpet or mattresses). Heatmap analysis (SPSS) was performed to elucidate the similarities/differences difference of microbiome/resistome for HDM and mite samples. The compositions of the microbiome and resistome in dust and dust mite samples were examined using the *vegan* package (R). Nonmetric multidimensional scaling (NMDS) analysis was conducted using the Bray–Curtis dissimilarity index, employing the metaMDS function. Additionally, the *vegdist* function was used to obtain Bray–Curtis dissimilarity indexes for the microbiome and resistome matrices of the dust mite and dust samples. Mantel's test and Spearman’s rank correlation were applied to the Bray–Curtis dissimilarity indexes for inter- and intra-microbiome and resistome analyses.

### Ethical approval

The Research Ethics Committee (RES) of University of Galway approved the study (RES approval 19-Apr-02).

## Data Availability

Microbiome sequence data that support the findings of this study have been deposited in the National Center for Biotechnology Information SRA with the accession code PRJNA1111722: https://www.ncbi.nlm.nih.gov/sra/PRJNA1111722.
